# Chronic Kidney Disease—How Does It Go, and What Can We Do and Expect?

**DOI:** 10.3390/biomedicines11030977

**Published:** 2023-03-22

**Authors:** Li-Yun Chang, Jer-Ming Chang

**Affiliations:** 1 Division of Nephrology, Kaohsiung Medical University Hospital, Kaohsiung Medical University, Kaohsiung 807377, Taiwan; 2 School of Medicine, College of Medicine, Kaohsiung Medical University, Kaohsiung 807377, Taiwan

Chronic kidney disease (CKD), as a worldwide threat to public health, is a key determinant of poor health outcomes, but the severity of the problem is probably not fully appreciated [1]. CKD has no cure, but depending on the underlying causes, some clinical parameters can be properly managed, consisting of measures to help control the signs and symptoms, reduce complications and slow the progression of the disease. CKD usually progresses slowly, but it is frequently accompanied by concomitant metabolic and age-related factors which might aggravate its progression. Human life succumbs to failed organs, but patients with end-stage kidney disease (ESRD) may not. Renal replacement therapy helps to extend their lives and retrieve a certain quality-of-life level. However, the advantages come along with unmeasurably increased medical expenses even in developed countries, and, therefore, we know to study CKD pathogenesis and develop strategies to hinder its progression. Several valuable works, studying various aspects of the pathogenesis and management of CKD, have been collected in this Special Issue of *Biomedicines*.

Polycystic kidney disease and congenital anomaly of the kidney and urinary tract are the most commonly seen genetic/congenital disorders leading to CKD. However, past studies have shown that ADTKD (autosomal dominant tubulointerstitial kidney disease) may not be an uncommon type of CKD caused by heterozygous mutations [2]. *UMOD*, encoding uromodulin, was the first identified mutation and the most common cause of ADTKD. The prevalence of ADTKD-UMOD remains unclear, and recent studies have shown that it represents up to 0.3~2% of all CKD patients [3]. Chen et al. studied 228 CKD individuals in Taiwan via exome sequencing and they uncovered three *UMOD* missense variants, of which two had not been previously reported. The abnormal functional expression of the mutated *UMOD* protein was also demonstrated in HEK 293 cell culture. They concluded that ADTKD-UMOD might represent a small but significant (1.31%) cause of CKD. Multi-center screening would provide a better understanding of the mutation landscape in Taiwan and contribute to the ADTKD research field.

Immunoglobulin A nephropathy (IgAN) is the most common pathology noted in all primary glomerulonephritis (estimated to be at least 30%), and a multi-hit theory has been proposed by researchers [4]. The association between IgAN and the functional mucosal systems has been extensively studied, as IgA represents the major secretory immunoglobulin from mucosa, and such an association was actually substantiated by the favorable clinical responses of tonsillectomy and the targeted release formulation of budesonide in IgAN patients. Human mucosal systems constantly exposed themselves to exogenous antigens and microbes, and did not elicit specific immune responses in most individuals. It remained unclear how the interactions between mucosal systems and exogenous stimulations might induce the expression of IgA1 containing galactose-deficient O-glycans in its hinge region (galactose-deficient IgA1; Gd-IgA1). Kano et al., in a review, discussed the dysregulated mucosal immune response in IgAN, specifically focused on gut-associated and nasal-associated lymphoid tissue.

CKD is very commonly complicated by various comorbid illnesses, especially those with metabolic derangement. Good control of these comorbidities can often help to lessen CKD severity and its progression. Numerous studies have suggested goals of control for individual diseases, most notably for diabetes mellitus (DM) and hypertension. However, the applicability of these goals in CKD patients is somewhat questionable because their outcomes would be affected by not just one but many other factors. A good example is the application of HbA1c < 7% as a commonly accepted marker of good glycemic control. The level of HbA1c is affected by the half-life of red blood cells, the level of hemoglobin and other factors. CKD patients have shortened red blood cell half-lives and lower hemoglobin levels, unfavorably contributing to the formation of HbA1c and therefore its applicability as a stable marker of glycemic control [5]. Longitudinal follow-up studies have suggested that HbA1c 7~9% [6,7] might be a safe and proper target for CKD stage 3 and 4 (but not 5) patients to slow the progression to ESRD. Global clinical practice guidelines have also revised their suggestion to not have too strict a glycemic target for diabetic CKD patients [8]. It is unclear whether HbA1c and other components of metabolic syndrome could play a role in non-diabetic CKD patients. In this issue, Hung, Su and Niu et al. analyzed the role of HbA1c, metabolic syndrome, obesity-related indices and hyperuricemia in the prediction of renal function deterioration in non-diabetic individuals with or without CKD. They concluded that inadequate control of these above metabolic factors was also associated with worse kidney outcomes in the absence of diabetes.

There is no panacea to cure CKD, even though researchers had devoted efforts toward it and clinical studies have shown promising results. Owing to its complexity, CKD management is still an unaccomplished aim. The development and clinical application of sodium-glucose cotransporter 2 inhibitor (SGLT2i) has led to a paradigm shift in the management of diabetic patients to benefit their cardiovascular and renal outcomes [9]. The extent of the organ protection is likely to make SGLT2i the most impactful drug class since the discovery of renin–angiotensin system inhibitors. SGLT2i has also been shown to slow CKD progression in non-diabetic patients with varying degrees of proteinuria. The study to evaluate its potential effect in the advanced CKD stages (stage 4/5) is ongoing [10]. Another route to tackle kidney failure is through a reduction in intestinal absorption of the small molecule toxins. Advanced CKD patients exhibited significant gut dysbiosis. An oral absorbent AST-120 (Kremezin^®^) was shown to reduce the absorption of the gut-derived uremic toxins (for example, indoxyl sulfate and p-cresol) and improve kidney histology in animal studies [11]. It was also shown to change the compositional and functional aspects of gut microbiota, but this phenomenon remained unclear in humans. Hsu et al. studied the effect of AST-120 in CKD patients and showed that it could partially restore the compositions and functional integrity of gut microbiota and introduce an imprint in their short- and medium-chain fatty acids’ metabolism. Further studies will be necessary to demonstrate the proposed benefits of AST-120 in terms of improving the gut microenvironment. A forward-looking study [12] has demonstrated the effects of the *Lactobacillus* mixture in reducing kidney injuries by down-regulating gut-derived uremic toxin production. It is promising that nephrology practitioners might have the chance to prescribe “kidney probiotics” to CKD patients in the future.

Nevertheless, physicians should always pay attention to avoiding the unwanted adverse effects derived from treatment, if there are any. Nephrologists have treated renal anemia with erythropoietin over the past few decades, and have greatly reduced the need for blood transfusions and therefore transfusion-associated diseases. Besides the correction of anemia, erythropoietin might improve cardiac function by binding to a receptor on cardiomyocytes [13]. However, Mochino et al. performed a multi-center, cross-sectional, observational study on 377 hemodialysis patients, and found that the serum levels of the anti-erythropoietin antibody (present in 4.5% of participants) were associated with a greater left ventricular mass index and a higher chance of inadequate systolic function, which are commonly used markers of left ventricular dysfunction. The causal relationship cannot be concluded from such a cross-sectional study, but their finding may remind all nephrology practitioners of drug-related safety issues.

Kidney transplantation has been recognized to be the best and the ultimate resolution for patients with advanced CKD. However, we have to admit that transplantation would push back the advanced CKD stage to stage 3a (one transplanted kidney), which is still a CKD stage. There is no doubt that kidney transplantation greatly improves patient outcomes and life quality. However, transplantation itself is a risk factor for diabetes development due to chronic exposure to certain immunosuppressive agents, even in non-diabetic CKD patients. Lim et al. indicated that patients after kidney transplantation were actually in a CKD stage with an unavoidable risk of diabetes incidence, and they discussed the pathogenesis and prevention strategies in this Special Issue.

As aforementioned, CKD is a serious threat to public health and is a never-ending story, even after kidney transplantation. Everyone in the nephrology field will have to participate and contribute continuous efforts to tackle this great challenge related to the pathogenesis and treatment of CKD.
